# Mineral nutrient dynamics in pecans (*Carya illinoensis*) ‘Mahan’ grown in southern China

**DOI:** 10.3389/fpls.2022.1003728

**Published:** 2022-10-28

**Authors:** Xiaodan Zhang, Jun Chang, Huadong Ren, Yaopeng Wu, Mei Huang, Shuang Wu, Shuiping Yang, Xiaohua Yao, Kailiang Wang

**Affiliations:** ^1^ College of Resources and Environment, Southwest University, Chongqing, Beibei, China; ^2^ Research Institute of Subtropical Forestry, Chinese Academy of Forestry, Hangzhou, Fuyang, China

**Keywords:** Pecan, mineral nutrient, nutritional characteristics, dynamic changes, fertilization recommendations

## Abstract

It is of great significance to study the nutritional characteristics of plants. Further understanding of plant mineral nutrient dynamics can provide theoretical basis for scientific fertilization to improve fruit quality and yield. In this study, eight mineral elements (N, P, K, Ca, Mg, Mn, Zn, B) were measured at regular intervals in leaves and kernels of the pecan “Mahan” planted in southern China. The study discussed the characteristics of mineral nutrient dynamics of pecan through the indicators of concentration, accumulation and cumulative relative rate, a new first proposed indicator, and focused on critical time, intensity, amount of mineral nutrients required in pecan during the fruit developing period, as well as the transfer information of the elements in leaves and kernels. The results show that the mineral nutrient requirements of the leaves and kernels are not identical, with an upward trend in nutrient accumulation within the kernel. The most abundant mineral nutrients in the leaves and kernels were N, K and Ca with Ca being greater than N in leaves. In particular, the concentration of Mn in pecan ‘Mahan’ is higher than that of other plants, and its Mg content is also higher than that of P in kernels. The dynamic changes of mineral nutrients in walnut showed obvious stages, with a trend of “slow (before mid-July) - fast (mid-July to late August) - slow (late August to late September) - fast (late September to harvest)”. The “critical period” of kernels was before mid-July, during which the cumulative relative rates increased rapidly, indicating that the kernels had a great potential to absorb mineral nutrients. Significant accumulation of mineral nutrients occurred from mid-July to late August and late September to the end.

## Introduction

Mineral elements are essential nutrients for plants and play a vital role in their growth, being an indispensable fundamental for yield and quality (Lu, 2007; Fu et al., 2021; Jovovic et al., 2021). The nutritional characteristics of different crops vary. Understanding the dynamics of mineral nutrients in crops is one of the necessary conditions to grasp the nutritional characteristics of crops (Mir et al., 2015), which is also important to achieve precise, efficient, green and safe fertilization. Today, the nutrient dynamics of vegetables and food crops have been studied to some depth worldwide (Hocking, 1994; Gallardo et al., 2021), and some publications have also been prepared on this aspect of common fruit trees (Kim and Wetzstein, 2005; Stateras and Moustakas, 2018). Pecan is an oil-nut deciduous tree species introduced in China in recent years with good economic and ecological benefits, and its nuts are hugely popular in the Chinese market (Zhang et al., 2015). In addition to being used for the production of edible nuts, pecan is also one of the high-quality oilseed species with great potential for exploitation as an oil supply (Zhang et al., 2022), but it has been cultivated for a short period of time in China and on a small area, and has not received enough attention yet. To further explore the nutrient requirements of pecan tree and to effectively develop the production potential of pecan in China, it would be useful to study the internal plant dynamics of mineral nutrients.

There are significant phenological characteristics of mineral nutrients in fruit trees (Cruz et al., 2019), which have been demonstrated in apple, peach, citrus, olive, kiwi and walnut species (Clark and Boldingh, 1992; Kim and Wetzstein, 2005; Stateras and Moustakas, 2018). In some early efforts of pecan, Cresswell observed that the leaf N, Pand K concentrations varied with time, gradually decreasing and Ca, Mg and B concentrations changing in opposite trends (Cresswell and Wickson, 1986). The results of the Worley were consistent with Cresswell, with only Mg found to be less variable (Worley, 1977). Kim measured both the concentrations and accumulation of mineral nutrients in pecan leaves and found that they did not follow exactly the same trend over time (Kim and Wetzstein, 2005). By measuring the leaves and kernels of ‘Bonny’ pecan, Jia found that the mineral nutrients in the kernels showed drastic changes between 95 and 125 days of fruit development, and leveled off in the leaves and kernels after 125 days (Jia et al., 2016). Shen observed the dynamics of mineral nutrients in the fine roots, fruiting branch leaves, male inflorescences and fruits of ‘Mahan’ and found clear patterns of micronutrients element concentrations in different organs with the change of fruit developing period (Shen et al., 2017). However, these studies had mostly focused on pecans in their origin and only addressed the concentration dynamics of certain periods and nutrients, with less attention paid to trees grown in China, and a lack of understanding of mineral nutrient accumulation dynamics over the fruit developing period. In this study, the leaves and kernels of pecan ‘Mahan’ will be regularly sampled, measured and analyzed to reveal the nutritional characteristics of pecan variety, and to provide some reference for rational fertilization from the perspective of dynamic changes in mineral nutrient concentrations and accumulation in leaves and kernels (Rey et al., 2006; San-Martino et al., 2010).

## Materials and methods

### Plant materials and their origins

The material tested in this study was 14-year-old pecan(*Carya illinoensis*) varieties of ‘Mahan’ (six), which grew well and were free of pests and diseases. The pecan experimental forest is located in Hongzhai, Jiande, Zhejiang (29°28′N, 119°23′E). The area has an elevation of 100 m, with average annual temperature and accumulated rainfall of 16.7°C and 1500 mm, respectively. The pecan trees planted in purple soil with pH of 5.39. According to a previous chemical analysis of the soil, the following results were found (mg·kg^-1^): 908 total N, 14.8 Olsen-P, 98 available-K, 872 exchangeable Ca^2+^, 5550 Mg^2+^, 405 Mn^2+^, 27.9 Cu^2+^, and 61 Zn^2+^.

Nutrient changes in pecan kernels introduced to China were more pronounced between 95 and 145 days of development than at other times of the year (Jia et al., 2016). In this study, a weekly sampling was taken from 25 August and 6 October, and each fortnight thereafter. We collected samples on 10 May, 25 May, 10 June, 27 June, 9 July, 25 July, 10 August, 25 August, 1 September, 8 September, 15 September, 22 September, 29 September, 6 October and 18 October in 2017. 100 complete leaves and 0.50 kg of fruits were collected in four directions (east, west, south and north) in the middle of the canopy at each time. Sampling was completed and returned to the laboratory in time for processing.

### Sample determination

The plant samples were washed with tap water and deionized water, then dried in an oven at 105°C for 30 min, then dried at 75°C to a constant mass and crushed through a sieve (sieve: 0.25 mm). The kernels were weighed before and after drying using an electronic balance with an accuracy of 0.01.

Place the sample (0.2g, accurate to 0.0001g) in a PTFE digestive tube, add 3mL nitric acid and 2mL hydrogen peroxide. The digestive tube was placed on the acid furnace at 150°C (sealed), and then placed in the microwave digestion device. The microwave digestion procedure was shown in **Table 1**. After digestion, the digestive tube was removed and cooled, and the volume was fixed to 25mL with pure water. The treatment without sample in the same batch was blank control. The mineral elements in the digestion solution were determined by inductively coupled plasma mass spectrometer (PerkinElmer NexIon 300D, USA) and inductively coupled plasma spectrometer (Thermofisher iCap7400, USA), and the elements were accurately quantified by multi-element mixed standard solution.

**Table 1:** Microwave digestion procedure.

Step	Temperature/(°C)	Ramp time/(min)	Hold time/(min)
1	130	15	10
2	200	10	20

### Data process

Microsoft Excel.2019 was used to analyze and plot the data collected. Mineral nutrient indicators: cumulative increment, cumulative rate of increase, cumulative contribution rate, and cumulative relative rate data were calculated in accordance with the formula.


$$\begin{array}{l} \text{Cumulative increment}/(10^{-3}\cdot {\text{g}}^{-1})\\ \;=\text{cumulative at the next time}\\ \;-\text{cumulative at the previous time}\\ \text{Cumulative rate of increase}/(10^{-3}\cdot {\text{g}}^{-1})\\ \;=\text{cumulative increment}\times 7/\text{number of days in the period}\\ \text{Cumulative contribution rate}/(\%)\\ \;=\text{cumulative increment}\times 7\times 100/\\ \;(\text{number of days in the period}\times \text{total cumulative volume})\\ \text{Cumulative relative rate }=\;(\text{cumulative rate of increase}\\ \;\text{in the next period }-\text{cumulative rate of}\\ \;\text{increase in the previous period})\;/\text{ | Cumulative}\\ \;\text{ volume growth in the next period }| \end{array}$$

## Results

### Dynamics of kernel weight

**Figure 1:**
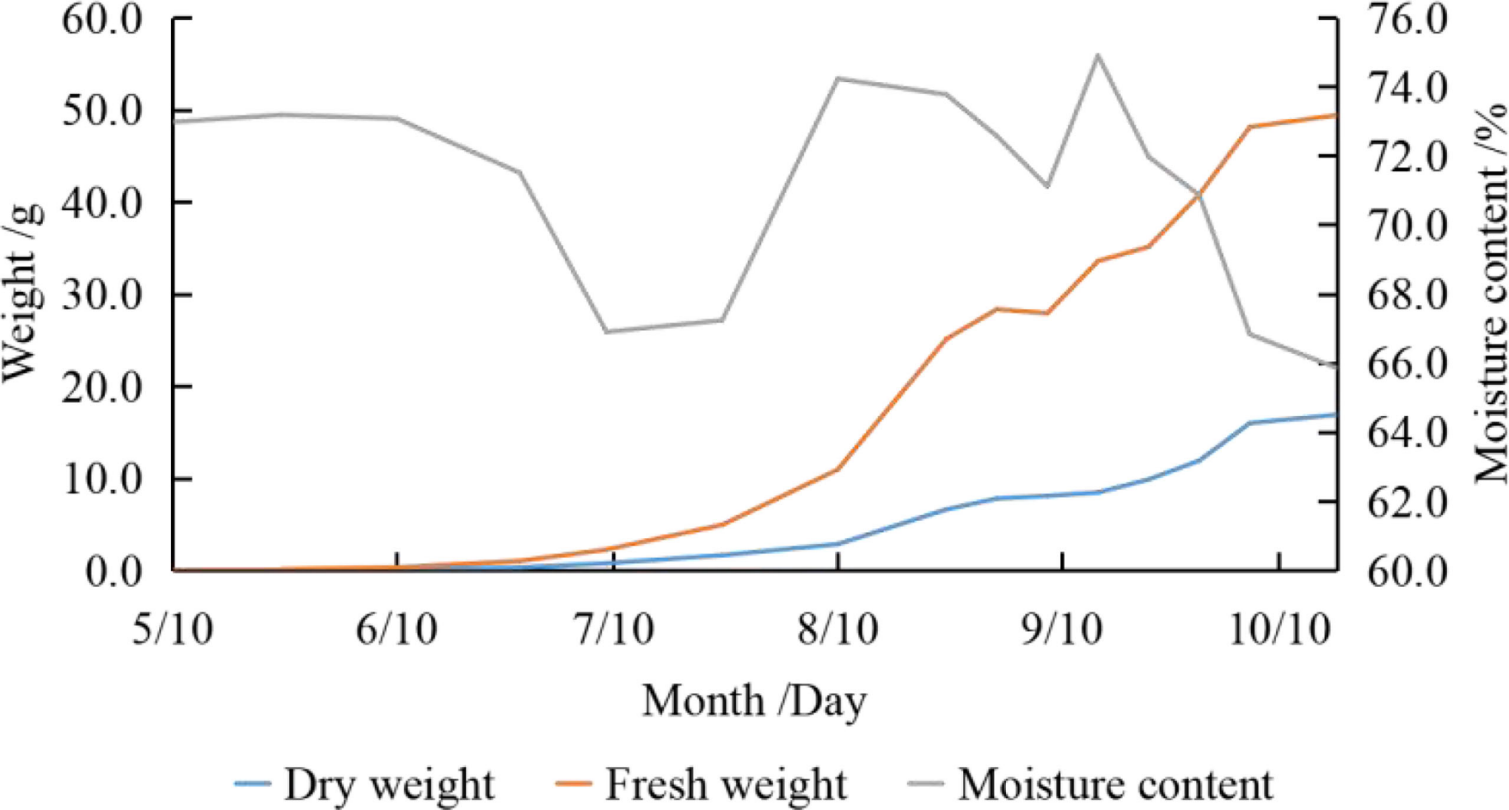
Dynamic changes of kernel weight.

Both fresh and dry kernel weight showed an increasing trend with slight fluctuations (**Figure 1**). The fresh kernel mass per fruit rose from the beginning (0.02g) to the end (52.38g) of the fruit developing period, with a high rate of increase (3.082g·W^-1^ to 6.616g·W^-1^) in mid-late August and late September to the end (weekly), contributing 33.22% and 32.99%, respectively. The trend in dry mass per fruit was similar to that of fresh mass, which increased from the beginning of the fruit developing period (5.19×10^-3^g) to the end (17.36g). The dry mass per fruit increased significantly (1.187g·W^-1^ to 2.878g·W^-1^) in mid-late August and from mid-September to the end of development, contributing 28.54% and 51.49%, respectively.

Moisture is a large proportion of the fresh mass. The trend of water content was “down - up - down” (66.85% to 74.90%), with high levels in mid-May, mid-August and mid-September, and low levels in early July and the end of the fruit developing period.

### Dynamics of mineral nutrient concentrations

**Figure 2:**
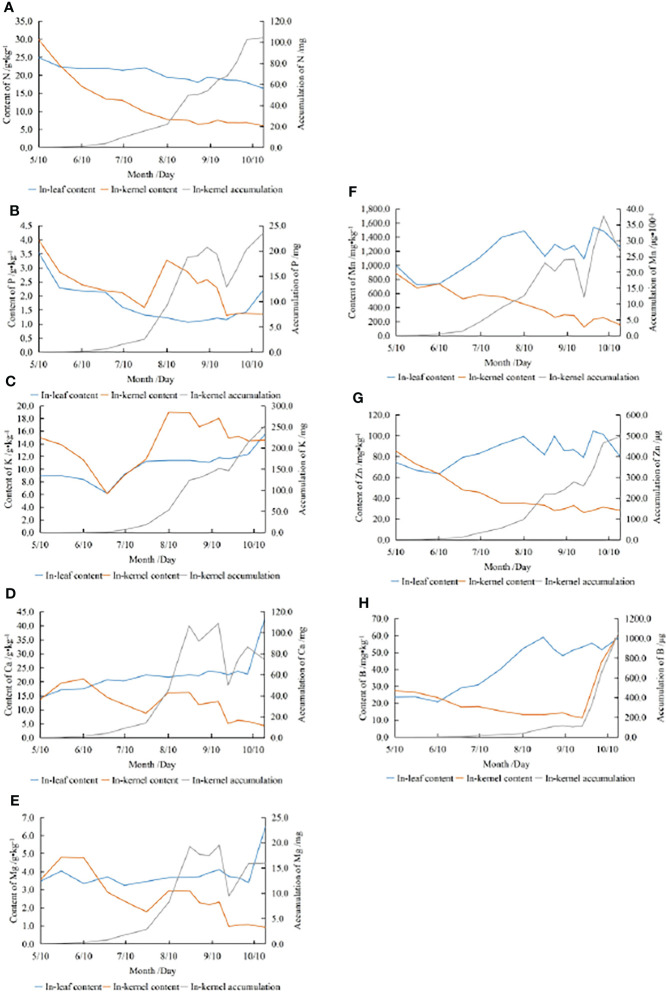
Dynamics of the concentrations and accumulation of N **(A)**, P **(B)**, K **(C)**, Ca **(D)**, Mg **(E)**, Mn **(F)**, Zn **(G)**, B **(H)**.

The dynamics of the different mineral nutrients concentrations differs, and there are also significant differences in nutrient concentrations between leaves and kernels (**Figure 2**). The N concentrations in both leaves and kernels showed a continuous decreasing trend with slight fluctuations. From the beginning of the fruit developing period, N in leaves decreased slightly (24.93 g·kg^-1^ to 16.35 g·kg^-1^), with a decrease of 34.44% (**Figure 2A**). N in kernels decreased sharply from 29.90 g·kg^-1^ to 6.02g·kg^-1^, with a decrease of 79.87%. The average N concentration in leaves and kernels was 20.09 g·kg^-1^ and 11.26 g·kg^-1^, respectively.

The P concentration in the leaves decreased first and then increased, reaching the maximum value (3.54 g·kg^-1^) in the beginning of the fruit developing period and the minimum value (1.07 g·kg^-1^) in late August. P in the kernels decreased from the highest level (3.99 g·kg^-1^) and fluctuated considerably from late July to late September, and reached the minimum value (1.31 g·kg^-1^) in late September. The average P concentration in leaves and kernels was 1.66 g·kg^-1^ and 2.26 g·kg^-1^ respectively (**Figure 2B**).

Before August, the K concentration in leaves and kernels had a similar trend, which decreased first and then increased, and reached the minimum values in late June (6.16 g·kg^-1^ and 6.11 g·kg^-1^). After July, K in the leaves increased slowly and reached the maximum value (15.54 g·kg^-1^) at the end of the fruit developing period. K in kernels increased rapidly and reached the maximum value (18.98 g·kg^-1^) in mid-August, and decreased slightly after late August. The overall increase of K in leaves was 73.67%. K in kernels varied greatly, but there was no significant difference between K concentration at the end of the fruit developing period and that at the beginning. The average K concentration in leaves and kernels was 10.76 g·kg^-1^ and 14.41 g·kg^-1^ respectively (**Figure 2C**).

The trends in Ca and Mg concentrations were similar, with an overall trend of increasing in the leaves and fluctuating downwards in the kernels. Ca and Mg increased steeply in leaves in October (increase of 86.45% and 91.31%, respectively) and fluctuated considerably in kernels from late July to late September. The Ca concentration in the leaves increased continuously from the beginning of the fruit developing period (14.15 g·kg^-1^) to the end (42.33 g·kg^-1^), with an increase of 199.0%. From mid-June (21.00 g·kg^-1^) to the end of the fruit developing period (4.29 g·kg^-1^), Ca in kernels increased slightly at first and then decreased, with a decrease of 68.55%. The average Ca concentration in leaves and kernels was 22.46 g·kg^-1^ and 12.01 g·kg^-1^, respectively. Mg in leaves changed gently before October, and it varied from 3.24 g·kg^-1^ to 6.50 g·kg^-1^ during fruit development, with an increase of 87.58%. Mg in kernels fluctuated from late May (4.80 g·kg^-1^) until the end of the fruit developing period (0.92 g·kg^-1^), with a decrease of 74.05%. The average Mg concentration in leaves and kernels was 3.85 g·kg^-1^ and 2.45 g·kg^-1^ respectively (**Figures 2D, E**).

Mn and Zn concentrations increased in the leaves, while fluctuating downed in the kernels. Mn in leaves decreased slightly at the beginning of fruit growth, increased sharply from late May(minimum value 0.72 g·kg^-1^) to late July, and showed a high fluctuation from late July to late September (maximum value 1.54 g·kg^-1^), and decreased slightly after September. Mn in leaves increased by 26.60%. Mn in kernels decreased from the beginning of the fruit developing period (0.89 g·kg^-1^) to late September (0.12 g·kg^-1^), and then showed little fluctuation. Mn in kernels decreased by 82.82%. The average Mn concentration in leaves and kernels was 1.18 g·kg^-1^ and 0.42 g·kg^-1^ respectively. Zn in the leaves increased significantly from mid-June (63.40 mg·kg^-1^) to late July, and reached the maximum (104.4 mg·kg^-1^) in late September, with an increase of 8.19%. Zn in kernels decreased from the beginning(85.15mg·kg^-1^) to late September (26.33mg·kg^-1^), with a decrease of 66.51% over the fruit developing period. The average Zn concentration in leaves and kernels was 85.13mg·kg^-1^ and 41.66mg·kg^-1^ respectively (**Figures 2F, G**).

The trend of B in leaves was similar to that of Mn and Zn. B concentrations increased significantly from mid-June (20.75 mg·kg^-1^) to late August (59.03 mg·kg^-1^), and then decreased slightly, increasing 148.36% during fruit development. B in the kernels decreased slowly from the beginning to late September (minimum 11.40 mg·kg^-1^) and then increased significantly until harvest (60.60 mg·kg^-1^), with an overall increase of 120.97%. The average B concentration in leaves and kernels was 43.33 mg·kg^-1^ and 22.68 mg·kg^-1^ respectively (**Figure 2H**).

### Kernel mineral nutrients accumulation

Nutrient concentration is valuable in assessing whether a plant is well-nourished, but concentration alone does not reflect the net movement of nutrients. The dynamics of mineral nutrient accumulation in this study did not always coincide with that of the concentration (**Figure 2**). The accumulation of all eight mineral elements in the kernels showed an increasing trend, with a more rapid increase from mid-July to late August and from late September to the end than in other periods. N, K and Zn showed a continuous increase with slight fluctuations. The dynamics of P, Ca, Mg and Mn accumulation fluctuated considerably, rising sharply from late July to late August, followed by a ‘plateau’ between late August and mid-September, then a significant decline from mid-September to late September, and finally a downward trend in Ca and Mn accumulation prior to harvest. The accumulation of B increased slowly until late September and then increased significantly before fruit harvest. From late September to the end, B accumulation was 0.94 mg, accounting for 89.33% of the total cumulative increment at the end of the fruit developing period for this element. At the end of fruit growth, the order of elements accumulation in individual fruit was K (0.25g) > N (0.10g) > Ca (0.07g) > P (23.48mg) > Mg (15.95mg) > Mn (2.64mg) > B (1.05mg) > Zn (0.50mg).

The cumulative relative rate is the ratio of the increase rate of mineral nutrient accumulation at one stage of growth compared to the previous stage, and the cumulative contribution rate reflects the ratio of the cumulative increase at one stage to the final accumulation of total fruit (**Table 2**). The cumulative relative rates of all mineral nutrients were greater from late May to early July, followed by late July to late August and September. Between 15 September and 22 September, the cumulative relative rates for all elements except B were negative. And cumulative relative rates for each element were also negative in mid to late August and early to mid-October. Contributions of mineral nutrient accumulation above 10% were concentrated in mid to late August, with P, Ca and Mg accumulation also above 10% from late July to mid-August, followed by late September to early October.

**Table 2:** Cumulative relative rate and cumulative contribution rate of 8 mineral elements over time.

Mineral elements	N		P		K		Ca		Mg		Mn		Zn		B	
Periods	CRR	CCR	CRR	CCR	CRR	CCR	CRR	CCR	CRR	CCR	CRR	CCR	CRR	CCR	CRR	CCR
Before 5/10	0	0	0	0	0	0	0	0	0	0	0	0	0	0	0	0
5/10-5/25		0.17	0	0.09		0.05		0.24		0.28		0.2		0.12		0.02
5/25-6/10	91.32	0.33	143.19	0.22	112.8	0.1	183.58	0.69	156.27	0.71	238.18	0.68	141.14	0.29	133.99	0.05
6/10-6/27	199.54	0.98	236.49	0.75	37.77	0.13	94.8	1.34	59.42	1.13	107.45	1.41	150.25	0.71	150.84	0.12
6/27-7/9	234.65	3.27	211.05	2.34	736.2	1.12	176.01	3.71	204.3	3.43	341.22	6.22	232.08	2.37	269.31	0.46
7/9-7/25	-20.96	2.58	-20.41	1.86	86.09	2.09	-16.7	3.09	-10.33	3.07	22.34	7.61	-14.5	2.03	5.89	0.49
7/25-8/10	0.71	2.6	564.35	12.36	186.14	5.99	492.15	18.28	384.62	14.9	-18.68	6.19	83.08	3.71	7.29	0.52
8/10-8/25	372.43	12.28	54.37	19.08	118.63	13.1	110.89	38.56	114.49	31.95	196.43	18.36	203.85	11.28	321.85	2.2
8/25-9/1	-92.6	0.91	-95	0.95	-83.86	2.11	-150.54	-19.49	-129.53	-9.44	-153.81	-9.88	-97.53	0.28	-13.68	1.9
9/1-9/8	264.94	3.31	667.98	7.33	85.14	3.91	156.34	10.98	86.57	-1.27	238.39	13.67	1371.98	4.1	-56.73	0.82
9/8-9/15	183.69	9.4	-181.43	-5.97	23.23	4.82	9.06	11.97	1095.72	12.62	-96.34	0.5	86.92	7.67	-231.76	-1.08
9/15-9/22	-55.74	4.16	-361.17	-27.51	-142.3	-2.04	-765.37	-79.67	-599.04	-62.96	-9179.77	-45.49	-150.65	-3.88	168.33	0.74
9/22-9/29	215.76	13.14	152.71	14.5	757	13.4	141.17	32.8	131.51	19.84	230.05	59.16	518.73	16.27	2709.68	20.8
9/29-10/6	50.29	19.75	15.8	16.79	-2.53	13.06	-49.96	16.41	2.18	20.27	-35.68	38.05	55.62	25.31	49.74	31.15
10/6-10/18	-94.27	1.13	-51.69	8.11	-30.37	9.09	-157.87	-9.5	-98.69	0.27	-166.18	-25.18	-85.83	3.59	-30.01	21.8

Cumulative relative rate (CRR); Cumulative contribution rate / % (CCR / %).

## Discussion

We sampled pecans planted in China at regular intervals to make simultaneous observations on the dynamics of leaves and kernels mineral nutrients concentrations and accumulation, and further analyzed the nutritional characteristics of pecans, which may cause some differences from the test subjects and entry points of previous studies.

N concentration was at high level in leaves and kernels at the beginning of fruit growth and then decreased due to ‘dilution effect’, while the outward transfer of leaf N to support fruit synthesis of starch, protein and fatty acids was another reason for the decrease (Kim and Wetzstein, 2005; Rentsch et al., 2007). The increase in P concentration in the kernels from late July to early August broke the “dilution effect”. Since P is involved in the conversion of saturated fatty acids to unsaturated fatty acids later in fruit development, it can be assumed that the fruits were preparing for the conversion of fatty acid fractions (Chang et al., 2019a; Cao et al., 2013). In addition, P is a major component of the phosphorylated intermediates of energy metabolism, which may be an important reason for the vigorous demand for P during the high physiological activity of the fruits (Shen et al., 2011). The demand for K in leaves and kernels was significantly enhanced after late June, and it was hypothesized that K+ regulated the status of K+ uptake by roots through the formation of a demand signal (Drazeta et al., 2004; De Schepper et al., 2013). K fertilization of the tree is required at this time to meet the conversion and transport of sugars within the fruits and to support the rapid transport of photosynthetic products from the leaves to the kernels and conversion into oil (Chang et al., 2019b; Hunter and Hammer, 1956).

There was an overall upward trend in Ca and Mg concentration in the leaves, especially a significant increase in the pre-harvest period, which was associated with increased input due to transpiration and a decrease in fruit demand in the later stages of growth. However, it is interesting to note that Ca is a immobile element and Mg is highly mobile, while the phenomena are consistent for both. On the one hand, it could be speculated that Ca and Mg accumulation in leaves may be increased by different mechanisms, as Ca can be transferred to the leaves and fruits through the xylem but is difficult to transfer out through the phloem (Rogiers et al., 2000; Choat et al., 2009), which led to Ca accumulation in the leaves. In contrast, Mg accumulated heavily in both leaves and fruits in late growth, when fruit accumulation of Mg was sufficient (Nèjia et al., 2016) and Mg in leaves was higher and no longer transferred outwards in significant amounts, a phenomenon was also found in hickory (*Carya cathayensis*) (Xia et al., 2014). On the other hand, a decrease in Ca accumulation within the kernels was observed in mid-late September and early mid-October, suggesting a transfer of Ca from the kernels to the outside, which had the potential to transfer to the leaves, but was contrary to the immobility of Ca, a situation that needs further study.

Mn, Zn and B are all essential elements for tree growth (Alejandro et al., 2020; Matthes et al., 2020; Zeng et al., 2021) and the similar trends within the leaves suggest that there may be some correlation between the three elements in the nutritional and physiological activities of pecan, which needs to be further investigated. The high fluctuations in Mn and Zn concentrations in the leaves during the longer light hours in late July may be due to the fact that both are closely related to photosynthesis (Hansch and Mendel, 2009). Later in the growing season, the moisture content within the kernel decreased (i.e. the hydraulic conductivity from the xylem into the fruit decreased), which resulted in a decrease in the input of mineral elements dependent on xylem transfer, but a significant increased in B. It has been shown that there is an interaction between B and growth hormone (Matthes and Torres-Ruiz, 2016; Gómez-Soto et al., 2019), and it is speculated that the large increase in B within the kernels in this study may be related to growth hormone.

The proportions of mineral nutrients concentrations varied considerably between periods. By dividing the periods according to the phases of mineral nutrient changes, and using N as a reference (taken 100), it can be found that: 1) The proportions of N concentration in leaves and kernels were highest until mid-July, followed by Ca (53.4 and 59.8) and K (37.7 and 50.2); 2) Thereafter, the proportion of Ca concentration was higher in leaves (106-192) and K concentration in kernels (173-244) than in other elements; 3) Compared to other periods, the proportion of Ca concentration in leaves was highest at the end of the fruit developing period, and the proportion of K concentration in kernels was highest from late August to late August. In addition, the order of mineral nutrients concentrations within the leaves before harvest was Ca > N > K > Mg > P > Mn > Zn > B. The order within the kernels was K > N > Ca > P > Mg > Mn > B > Zn, which showed that N, K and Ca were dominant. Of these, Ca > N and Mg > P, as well as the higher Mn concentration (Lin et al., 2005; Hu et al., 2011; Xie et al., 2014), were more specific. These nutrient characteristics need to be taken into account when applying fertilizer. Mineral nutrients enter the soil after leaf drop and decomposition and re-enter the tree through the root system, while there is a loss of mineral nutrients after the fruits has been harvested (especially K), which is a key point to consider when applying fertilizer in the coming year.

The changes in the accumulation of the eight mineral elements in this study had similar characteristics, with a trend of “Slow (before mid-July) - fast (mid-July to late August) - slow (late August to late September) - fast (late September to harvest)”:1) Before mid-July, the accumulation of mineral nutrients in the kernels did not increase much, but the cumulative relative rates increased exponentially and rapidly, indicating that the kernels had an urgent need for nutrients during this period and had a large absorption potential, which was the “critical period”. If the supply does not meet the demand, the growth of the plant will be adversely affected. 2) From mid-July to late August, the accumulation of mineral nutrients increased significantly, with cumulative relative rates and cumulative contribution rates were high, which was the “period of heavy accumulation”. This is the period when the fruits needed to be well stocked with nutrients for later quality formation (Clark and Boldingh, 1992), and fertiliser applications must take this period of high nutrient requirement into account. 3) Changes in mineral accumulation were flat from late August to late September, a critical period for sugar conversion and fat synthesis (Jia et al., 2016); and since autumn tip growth was observed on the tree in late September, it was hypothesized that P, Ca, Mg and Mn, which had declined significantly in accumulation, were transferred from the kernels to the new tips. However, Chang showed that pecan shells was hardening in this time (Chang et al., 2019) and that Ca was involved in fruit cell wall formation (Hepler, 2005), so the reasons for the outward transfer of Ca need to be further investigated. 4) In late September until harvest, the cumulative relative rates and cumulative contribution rates were high, especially for B, which was also the period when nutrients were accumulated in large quantities. Scholars have classified the developmental periods of pecan from different perspectives (Dong, 2002; Worley, 2002). In our study, the periods of mineral element changes were basically consistent with the developmental stages classified by Dong Runquan according to fruit morphological changes (Dong, 2002), indicating that mineral nutrient accumulation and fruit morphology have some correlation.

## Conclusions

Our study showed that the dynamics of the eight mineral nutrients were not entirely consistent, with an overall decrease in N and P concentrations and an increase in other elements in the leaves, and an overall increase in K and B concentrations and an opposite trend in the other elements in the kernels. The proportions of mineral nutrients concentrations varied considerably in different periods, but all were dominated by N, K and Ca, with the highest proportion of Ca concentration in leaves and the highest proportion of K concentration in fruits.

The dynamics of mineral nutrient accumulation in the kernels showed an overall upward trend, with the highest K accumulation occurring at the time of fruit harvest. The elemental dynamics during the fruit developing period were all phased, with a trend of “Slow (before mid-July) - fast (mid-July to late August) - slow (late August to late September) - fast (late September to harvest) “, which largely overlaps with kernel mass changes. The “critical period” is from the start of fruit growth to late July, when the demand for mineral nutrients is urgent. The kernels needs to store large amounts of mineral nutrients from mid-July to late August to supply quality formation, and from late September to the end is also the period when nutrients enter the kernels in large quantities.

## Data availability statement

The original contributions presented in the study are included in the article/supplementary materials. Further inquiries can be directed to the corresponding authors.

## Author Contributions

JC, SY, and XZ proposed the idea. JC and XZ completed investigation and analyzed data. JC and SY managed projects. XY and JC provide resources. XZ made figures and wrote this paper. All authors participated in the experiments and reviewed the manuscript. All authors contributed to the article and approved the submitted version.

## Funding

The work was supported by the Fundamental Research Funds for the Central Non-Profit Research Institution of CAF (CAFYBB2020SY014), and the Zhejiang Provincial Academy Cooperation Forestry Science and Technology Project (2018SY04).

## Conflict of interest

The authors declare that the research was conducted in the absence of any commercial or financial relationships that could be construed as a potential conflict of interest.

## Publisher’s note

All claims expressed in this article are solely those of the authors and do not necessarily represent those of their affiliated organizations, or those of the publisher, the editors and the reviewers. Any product that may be evaluated in this article, or claim that may be made by its manufacturer, is not guaranteed or endorsed by the publisher.

## Abbreviations

CRR, Cumulative relative rate; CCR, Cumulative contribution rate.
